# Role of Host and Bacterial Lipids in *Pseudomonas aeruginosa* Respiratory Infections

**DOI:** 10.3389/fimmu.2022.931027

**Published:** 2022-07-04

**Authors:** Pamella Constantino-Teles, Albane Jouault, Lhousseine Touqui, Alessandra Mattos Saliba

**Affiliations:** ^1^ Department of Microbiology, Immunology and Parasitology, Faculty of Medical Sciences, Rio de Janeiro State University, Rio de Janeiro, Brazil; ^2^ Sorbonne Université, Centre de Recherche Saint-Antoine, Inserm, Institut Pasteur, Mucoviscidose et Bronchopathies Chroniques, Département Santé Globale, Paris, France

**Keywords:** lipid, respiratory infection, *Pseudomonas aeruginosa*, phospholipase, inflammation, virulence

## Abstract

The opportunistic pathogen *Pseudomonas aeruginosa* is one of the most common agents of respiratory infections and has been associated with high morbidity and mortality rates. The ability of *P. aeruginosa* to cause severe respiratory infections results from the coordinated action of a variety of virulence factors that promote bacterial persistence in the lungs. Several of these *P. aeruginosa* virulence mechanisms are mediated by bacterial lipids, mainly lipopolysaccharide, rhamnolipid, and outer membrane vesicles. Other mechanisms arise from the activity of *P. aeruginosa* enzymes, particularly ExoU, phospholipase C, and lipoxygenase A, which modulate host lipid signaling pathways. Moreover, host phospholipases, such as cPLA_2_α and sPLA_2_, are also activated during the infectious process and play important roles in *P. aeruginosa* pathogenesis. These mechanisms affect key points of the *P. aeruginosa*-host interaction, such as: i) biofilm formation that contributes to bacterial colonization and survival, ii) invasion of tissue barriers that allows bacterial dissemination, iii) modulation of inflammatory responses, and iv) escape from host defenses. In this mini-review, we present the lipid-based mechanism that interferes with the establishment of *P. aeruginosa* in the lungs and discuss how bacterial and host lipids can impact the outcome of *P. aeruginosa* respiratory infections.

## Introduction

*Pseudomonas aeruginosa* is a major etiological agent of both acute and chronic respiratory infections in immunocompromised and critically ill individuals. Several features explain the success of *P. aeruginosa* as an opportunistic pathogen, including the wide distribution of these bacteria in the environment (1, 2), the high frequency of multidrug-resistant strains (3–7), and the ability to produce an extensive and adaptable set of virulence factors, which are expressed depending on environmental conditions (8, 9).

In hospitalized patients, *P. aeruginosa* is usually associated with acute infections, representing one of the most common causes of hospital-acquired pneumonia (HAP) and the most isolated pathogen in ventilator-associated pneumonia (VAP) (10–12). Additionally, *P. aeruginosa* can persist in the lungs of individuals suffering from chronic respiratory diseases, such as cystic fibrosis (CF) or chronic obstructive pulmonary disease (COPD). In fact, *P. aeruginosa* is the most frequently detected and longest-lasting microorganism found in CF lungs, representing the main cause of morbidity and mortality for these patients (13–17).

The capacity to cause acute and chronic infections relies on the multifactorial nature of *P. aeruginosa* pathogenicity, which is supported by a wide range of proteins, carbohydrates, and lipids that allow colonization of abiotic surfaces and host cells, invasion of tissue barriers, killing of other bacterial species, and escape from the immune system. To highlight the role of lipids in the pathogenesis of respiratory infections caused by *P. aeruginosa*, this mini-review will focus on virulence mechanisms that use bacterial lipids or interfere with host lipids to favor the establishment and persistence of *P. aeruginosa* in the airways.

## Bacterial Lipids Acting as Virulence Factors

### Lipopolysaccharide (LPS)

pt?>LPS is composed of three domains: lipid A, the core oligosaccharide, and the O-antigen polysaccharide. *P. aeruginosa* lipid A consists of an acylated glucosamine disaccharide phosphorylated at the 1 and 4’ positions which can undergo several modifications, such as phosphorylation, hydroxylation, and addition of a palmitate acyl chain or aminoarabinose (18–22).

Lipid A is highly variable among *P. aeruginosa* isolates and also differs under planktonic and biofilm growth conditions (22, 23). Lipid A modifications are under the control of the two-component regulatory systems PhoP-PhoQ and PmrA-PmrB, which sense changes in environmental conditions and activate the expression of lipid A-modifying enzymes (20, 24, 25). In addition, PagL, which encodes a lipid A 3-O-deacylase, is particularly susceptible to mutations and is one of the hot spot loci detected in CF isolates (23). Mutations in PagL can lead to increased acylation of lipid A over time, with the penta-acylated lipid A seen in bacteria that initially colonize CF lungs being replaced by hexa- or, in the late stages of CF disease, hepta-acylated forms (26, 27).

During infection, lipid A modifications may confer greater resistance to cationic antimicrobial peptides or activate the inflammatory response (20, 25, 28, 29). It is interesting to note that the *P. aeruginosa* penta-acylated LPS binds TLR2 and is predominantly found in isolates from non-CF and early CF disease (30), whereas the hexa- and hepta-acylated forms that prevail in well-established *P. aeruginosa* infections, with higher acylation pattern been associated with higher CF disease severity in late stages, efficiently bind and activate the human TLR4-MD2-CD14 complex, inducing a more robust inflammatory response (31–34). Since CF individuals acquire *P. aeruginosa* infection from environment early in their lives, the inability to respond strongly to the penta-acylated LPS of environmental strains may facilitate the initial colonization of CF lungs by *P. aeruginosa*.

In mice lungs, TLR4 activation by *P. aeruginosa* LPS was able to induce NF-kB activation, secretion of proinflammatory cytokines and chemokines, and neutrophil recruitment, through a mechanism involving GM-CSF and the transcription factor PU.1 (35). It remains to be elucidated whether chronic exposure to *P. aeruginosa* lipid A contributes to CF morbidity by stimulating neutrophils to release mediators that promote lung damage or whether it induces LPS-hyporesponsiveness to reduce the inflammatory injury.

### Rhamnolipids

*P. aeruginosa* rhamnolipids are biosurfactants that consist of a dimer of fatty acids (3-(3-hydroxyalkanoyloxy) alkanoic acids - HAA), mainly composed of 10 carbon chains, linked to one or two molecules of L-rhamnose. The biosynthesis of rhamnolipids is under the control of various transcriptional and post-transcriptional regulators, with a critical role of the Rhl quorum sensing (QS) system that directly induces the transcription of the *rhlAB* operon and *rhlC*, which encode enzymes involved in HAA production and L-rhamnose transfer (36–38).

Rhamnolipids were first detected in sputum from CF patients chronically infected with *P. aeruginosa* (39), although a later study showed higher levels of rhamnolipids in *P. aeruginosa* isolates from intermittently colonized individuals than in isolates from chronically infected CF individuals (40). Curiously, when isolates from either chronic or acute infections were compared, a positive association between rhamnolipid production and acute infection was found (41).

In the airways, rhamnolipids favor the invasion of the epithelial barrier by *P. aeruginosa* and reduce bacterial clearance through innate immunity. On the respiratory epithelial surface, rhamnolipids slow down ciliary beat frequency and impair mucociliary transport, thus reducing the bacterial clearance (42, 43). Rhamnolipids initially interact with the apical membrane of epithelial cells and then progressively reach the basolateral membrane, displacing ezrin and disrupting the tight junctions, thus opening a paracellular route to invading bacteria (44). In the lungs, rhamnolipids inhibit phagocytosis by macrophages (45) and induce necrosis of neutrophils (46, 47), which play a key role in the defense against *P. aeruginosa*.

Several other effects related to rhamnolipid production may affect the respiratory infections caused by *P. aeruginosa*, since rhamnolipids can modulate swarming motility, participate in biofilm architecture by promoting the maintenance of channels that diffuse nutrients and oxygen, and mediate biofilm disruption by promoting the seeding dispersal of motile bacteria (48–51). Furthermore, rhamnolipids increase the bioactivity of the *Pseudomonas* quinolone signal (PQS) (52), a QS signaling molecule that controls several virulence factors (53), and can be detected in the lungs of CF patients infected with *P. aeruginosa* (54, 55). Importantly, rhamnolipids inhibit the growth of microorganisms that colonize CF lungs along with *P. aeruginosa*, such as *Staphylococcus aureus* and *Aspergillus fumigatus*, conferring them a competitive advantage in this environment (56–58).

### Outer Membrane Vesicles (OMVs)

Outer membrane vesicles (OMVs) are spherical nanoparticles with a lipid bilayer produced by blebbing of the bacterial outer membrane, containing a variety of lipids, sugars, DNA, RNA, and proteins. Depending on their content, which differs among *P. aeruginosa* strains (59, 60), OMVs can be involved in diverse biological processes, such as horizontal gene transfer (61–63), protection against phages (64), cell-cell communication (65), biofilm architecture (66, 67), antibiotic resistance (68, 69), escape from the immune system (70), and delivery of virulence factors into host cells (71).

The lipid membrane protects the vesicle content from extracellular degradative enzymes, enabling long-distance transport, and upon contact with host cells, fuses with cholesterol-rich host membrane microdomains known as lipid rafts, delivering their contents into the cell cytoplasm (72). The aminopeptidase PaAP, which is associated with the surface of OMVs from CF strains (59, 73), participates in the interaction with lung epithelial cells, optimizing the delivery of OMV content (73).

In *P. aeruginosa* respiratory infections, OMVs can release important virulence factors, such as the cystic CFTR inhibitory factor (Cif) (72). Cif decreases the apical membrane expression of CFTR and chloride secretion, altering mucociliary clearance (74), and inhibits TAP1, reducing MHC class I antigen presentation in the airways (75). OMVs are also associated with macrophage apoptosis (76) and can induce inflammation since they stimulate CXCL8 secretion by lung epithelial cells (59), as well as secretion of TNF-α, IL-6, MIP-2, CXCL1, CXCL-8, CCL2, IL-1β, and IFN-γ, and activation of the inflammasome in macrophages (77–79). Moreover, Park et al., 2013 showed *in vivo* that OMVs can cause dose-dependent pulmonary inflammation, with greater cellular recruitment and higher chemokine and cytokine secretion in mice lungs than in live bacteria (78). In contrast, release of sRNA by *P. aeruginosa* OMVs is associated with reduced LPS- and OMV-induced CXCL8 secretion by human airway epithelial cells along with decreased OMV-induced KC secretion in the bronchoalveolar fluid and reduced neutrophil recruitment in mouse lungs (80).

## Virulence Factors Targeting Host Lipids

### ExoU

ExoU, a phospholipase A_2_ (PLA_2_)-like enzyme that is injected into host cytosol by the type III secretion system machinery (81), is of special interest for acute respiratory infections caused by *P. aeruginosa*, since potent ExoU-mediated virulence is particularly associated with bloodstream invasion and increased morbidity and mortality in hospitalized patients, especially those suffering from HAP (70, 82–86).

ExoU and its chaperone SpcU are encoded in the PAPI-2 pathogenicity island (87–89), and are detected in about 20-40% of isolates of acute nosocomial infections, such as pneumonia and bacteremia (83–86, 90–92). A recent study performed with 243 isolates from *P. aeruginosa* bloodstream infection, including 50 with an *exoU*-positive genotype, showed that patients infected with *exoU*-positive strains had a higher proportion of respiratory infections, greater severity of illness, septic shock, and increased mortality compared with those infected with *exoU*-negative strains (85).

After injection into host cytosol, the ExoU C-terminal domain promotes localization of ExoU to the host cell membrane (93) through binding to the lipid phosphatidylinositol 4,5-bisphosphate (PI(4,5)P_2_) followed by conformational change and oligomerization of ExoU (94–96). Furthermore, both ubiquitin and PI(4,5)P_2_ binding is necessary for full ExoU PLA_2_ activity and cytotoxicity (96–98). Hence, although the N-terminal domain interacts with SpcU and has enzymatic activity, the C-terminal domain, which promotes ExoU-membrane lipid interaction, is also essential for ExoU-mediated virulence (81, 87, 99–102).

Animal models of acute pneumonia showed that, after infection, ExoU is rapidly expressed in mice lungs and that its levels increase over time (103). In these models, ExoU promotes a bacterial burden in the lungs, enhances dissemination of *P. aeruginosa* from the bloodstream to other organs, and reduces survival of infected mice (102–106).

PLA_2_ catalyzes the hydrolysis of the *sn*-2 position of membrane glycerophospholipids to release arachidonic acid (AA) and lysophospholipids, both potent lipid mediators. In the lungs, the ExoU PLA_2_ activity on membrane phospholipids generates free AA (107, 108) that is used to produce PGE_2_ (109, 110), whereas lysophospholipids (102, 110) produce PAF, which binds to PAFR in airway epithelial cells and activates NF-kB, stimulating a potent proinflammatory response characterized by secretion of CXCL8, as well its murine homologue KC, and a marked influx of neutrophils (109, 111, 112). However, ExoU kills neutrophils, as well as other phagocytic cells, causing a state of local immunosuppression that favors the persistence of ExoU+ and ExoU- bacterial strains (113–115).

Although ExoU injection causes reactive oxygen species (ROS) imbalance (116) and is cytotoxic for airway epithelial cells (102, 117), the remaining non-infected cells activate several transcriptional regulators, such as AP1 and NF-kB, modulating the host response (111, 112, 118, 119). Furthermore, the cytotoxic activity of ExoU also promotes endothelial cell damage, which is associated with ROS generation, membrane lipid peroxidation, and caspase-1 activation (107, 120). The ability to break down cellular barriers to bacterial dissemination, such as epithelium and endothelium, helps explain why ExoU is a predictor of invasive infections and has been associated with severe pneumonia followed by bacteremia and sepsis.

### Phospholipase C

*P. aeruginosa* synthesizes three types of phospholipases C (PLCs), the hemolytic PlcH, the non-hemolytic PlcN, and PlcB. All three PLCs hydrolyze phosphatidylcholine, the main component of cell membranes and lung surfactant, as well as other phospholipids found in eukaryotic membranes: PlcH also hydrolyzes sphingomyelin, PlcN targets phosphatidylserine, and PlcB, phosphatidylethanolamine (121, 122). To reach the extracellular medium, all three *P. aeruginosa* PLCs are secreted by the type II secretion system. However, to be transported across the inner membrane, PlcH and PlcN use the Tat system (123) whereas PlcB uses the Sec pathway (122). Furthermore, PlcH can be delivered into airway epithelial cells by OMVs (72).

In contrast to PlcN and PlcB, the role of PlcH in *P. aeruginosa* respiratory infections has been studied. Both intratracheal instillation of purified PlcH from *P. aeruginosa* and infection with a PlcH-producing strain, but not with its PlcH-defective isogenic mutant, were able to alter the respiratory mechanics during infection, with decreased pulmonary surfactant activity and impaired lung function (124).

Moreover, hydrolysis of phosphatidylcholine and sphingomyelin by PlcH yields diacylglycerol and ceramide, which are involved in signal transduction cascades that result in cellular processes such as cell death and inflammation (125, 126). Actually, intranasal administration of *P. aeruginosa* PlcH increases secretion of the proinflammatory cytokines and chemokines IL-6, IL-1β, TNF-α, MIP-1α, and MIP-2, as well as cellular infiltration, in mice lungs (127).

Despite the activation of the proinflammatory response, PlcH seems to favor *P. aeruginosa* persistence in the lungs (128). PlcH can increase the colonization of biotic and abiotic surfaces, since it contributes to *P. aeruginosa* attachment to CF bronchial epithelial cells and promotes biofilm formation on plastic when bacteria are grown in lung surfactant (129). In addition, PlcH is cytotoxic to macrophages (130) and suppresses the respiratory burst activity of human neutrophils (131), thus promoting bacterial survival in the lungs. Although PlcH is also cytotoxic to endothelial cells and inhibits angiogenesis (132), its role in bloodstream invasion remains to be determined.

The role of PlcN and PlcB in the pathogenesis of *P. aeruginosa* respiratory infections is unclear, although some properties can contribute to successful infection. Both PlcN and PlcB participate in the formation of *P. aeruginosa* biofilms (133), whereas PlcB is also associated with twitching motility (122).

### LoxA

Lipoxygenases play an important role in eukaryotic organisms since they metabolize polyunsaturated fatty acids (PUFAs), allowing the subsequent production of lipid mediators with strong immunomodulatory effects. Although lipoxygenases are rare in prokaryotes, Vance and colleagues reported in 2004 that *P. aeruginosa* secretes lipoxygenase A (LoxA), a functional homolog of the eukaryotic 15-lipoxygenase (134).

Lipoxygenase activity was detected in 34% of isolates from lungs of non-CF patients and in 18,3% of isolates from lungs of CF individuals, suggesting that LoxA may be secreted during *P. aeruginosa* respiratory infections (135). *In vitro* studies showed that, after interaction with host cell membranes and peroxidation of phospholipids, *P. aeruginosa* LoxA promotes biofilm growth on the surface of airway epithelial cells, helps bacterial invasion, and triggers arachidonoyl phosphatidylethanolamine-dependent ferroptosis (136–139). Furthermore, in a murine model of acute pneumonia, LoxA increased the production of the 15-LOX-dependent metabolites 13-hydroxy-octadecadienoic acid (13-HODE), 15- hydroxyeicosatetraenoic acid (15-HETE), and 17-hydroxydocosahexaenoic acid (17-HDoHE), which were then used to produce lipoxin A_4_ (LXA_4_), a bioactive lipid mediator with anti-inflammatory properties. Additionally, LoxA inhibited the secretion of the chemokines MIP-1α/CCL-3, MIP-1β/CCL-4, MIP-2/CXCL-2, CXCL-1, and KC in BALF, reduced the recruitment of inflammatory leukocytes, and promoted the persistence of *P. aeruginosa* in the lungs (135).

## Host PLA_2_ Enzymes and Their Role in *P. aeruginosa* Infection

**Figure 1:**
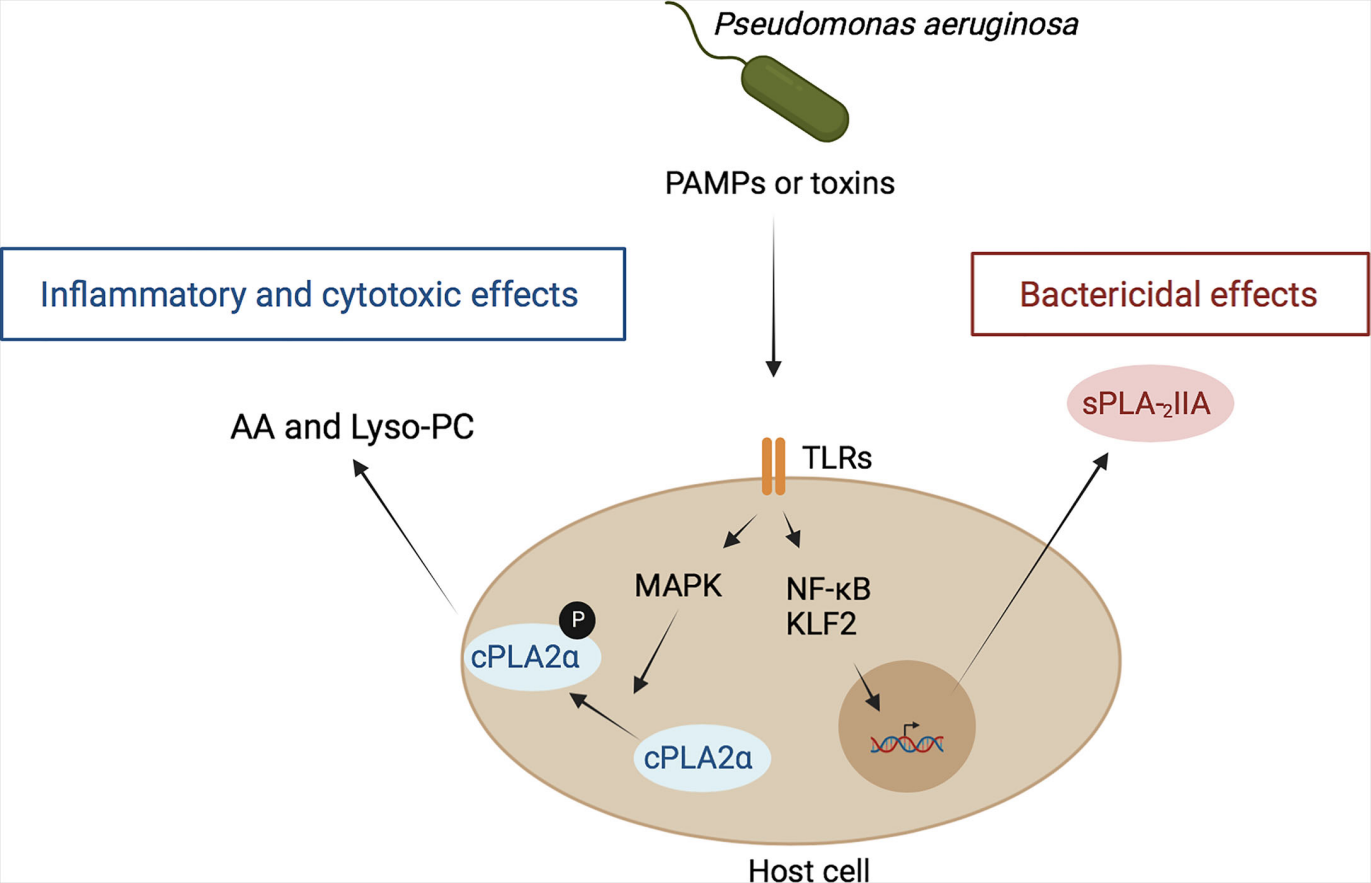
Role of host PLA_2_ in inflammation and antibacterial defense during *P. aeruginosa* infection. *P. aeruginosa* interacts with host cells through the binding of PAMPs of this bacterium, mainly LPS and flagellin to the host receptors TLR4 and TLR5, respectively, leading to NF-kB activation. In parallel, toxins, including ExoS, are also injected by this bacterium into host cells, leading to activation of the transcription factor KLF-2. Both processes result ultimately in the induction and secretion of sPLA_2_-IIA. Once in the extracellular media, this enzyme binds bacterial membranes and hydrolyzes their phospholipids leading to bacterial death (140). On the other hand, PAMPs stimulate cPLA_2_ translocation and activation *via* a MAPK-dependent mechanism. This leads to the hydrolysis of phospholipids of host cell membranes and subsequent release of free fatty acids, such as arachidonic acid (AA), and lysophospholipids, such as lysophosphatidyl-choline (Lyso-PC). AA is converted into pro-inflammatory eicosanoids and lyso-PC exert toxic effects on host cells (141, 142).

In addition to the PLA_2_ activity of *P. aeruginosa* ExoU, host cells also exhibit PLA_2_ enzymes that can mediate *P. aeruginosa*-induced toxicity (**Figure 1**). Among these enzymes, the host cytosolic PLA_2_α (cPLA_2_α), which hydrolyzes host membrane phospholipids releasing lysophospholipids and AA, plays a key role in *P. aeruginosa*-induced mouse mortality, mainly through cPLA_2_α-derived AA metabolites (141). In addition, it is likely that the accumulation of highly cytotoxic lysophospholipids, such as lysophosphatidylcholine, may participate in the deleterious effects of *P. aeruginosa*. This may indicate that cPLA_2_α represents a potentially interesting therapeutic target for the treatment of lung injury induced by *P. aeruginosa* infection and that a cPLA_2_α inhibitor can be used as a new strategy against inflammation.

Conversely, the host also produces a family of secreted PLA_2_ (sPLA_2_) that play a key role in defense against invading bacteria. For example, sPLA_2_-IIA can kill Gram-positive bacteria at very low concentrations (below 10 ng/ml), due to the unique preference of sPLA_2_-IIA for anionic phospholipids, such as phosphatidylglycerol (140), the main phospholipid component of bacterial membranes. In contrast, much higher concentrations (> 10 µg/ml) of sPLA_2_-IIA are required for its action on host cell membranes mainly composed of phosphatidylcholine, a poor substrate for sPLA_2_-IIA. The ability of sPLA_2_-IIA to kill Gram-negative bacteria, including *P. aeruginosa*, depends on factors that disrupt bacterial outer membrane organization, such as the bactericidal/permeability-increasing protein (BPI), which predisposes bacterial membranes phospholipids to sPLA_2_-IIA attack. Additionally, sPLA_2_-IIA can directly kill clinical isolates of *P. aeruginosa*, which chronically colonizes the upper airways of CF patients, but this effect is not affected by the high salt concentrations observed in CF secretions. Studies have shown that sPLA_2_-IIA kills a laboratory strain of *P. aeruginosa* and that sPLA_2_-IIA transgenic mice are protected from mortality by both laboratory and clinical strains of *P. aeruginosa* isolated from CF patients. These findings suggest that sPLA_2_-IIA may play a role in host defense during episodes of pulmonary infection by *P. aeruginosa* in CF patients (140).

## Discussion

**Figure 2:**
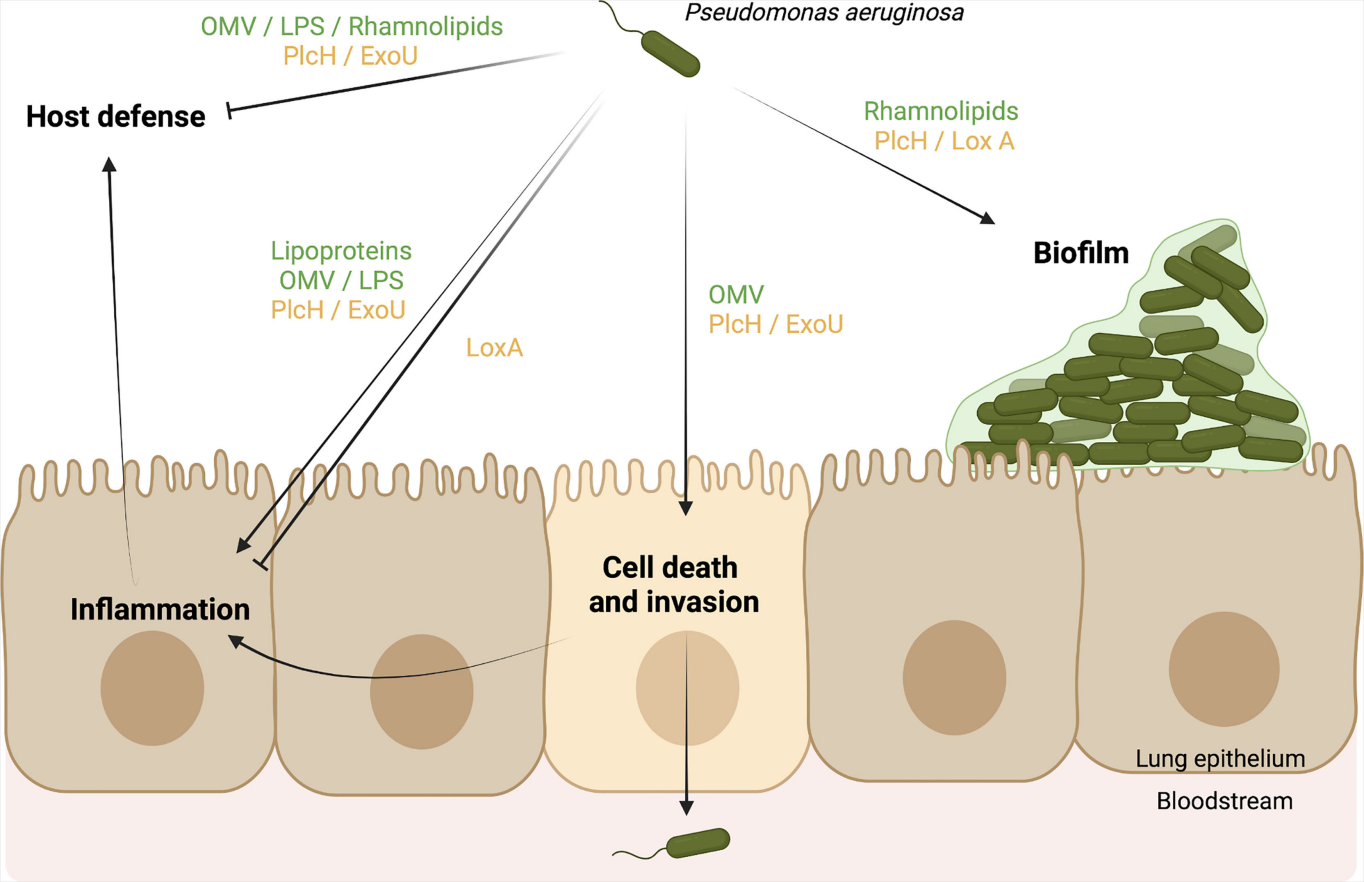
Scheme model of *Pseudomonas aeruginosa* virulence factors using a lipid-based mechanisms to cause respiratory infections. Bacterial lipids (green) and bacterial enzymes (yellow) targeting host lipids promote (→) or inhibit (˧) different biological functions during respiratory infections. They can (1) facilitate the biofilm formation of *P. aeruginosa*, (2) promote the death of host cells and the invasion of tissues, leading to the spread of *P. aeruginosa* in the bloodstream, (3) interfere with the inflammatory response, and (3) block the host defense (e.g. neutrophils, macrophages). As a result, *P. aeruginosa* can benefit from lipid mechanisms to persist in its host. Created in BioRender.com.

*P. aeruginosa* uses multiple virulence factors to cause acute and chronic respiratory infections. As summarized in **Figure 2**, *P. aeruginosa* lipids are able to exert important effects during infection. Bacterial lipids can protect *P. aeruginosa* from antibiotics and phagocytosis, promote bacteria-bacteria communication, provide a competitive advantage, participate in biofilm development, and interfere with the host response. In addition, host and bacterial lipid-modifying enzymes induced during the infectious process may promote the direct lysis of membranes and manipulate eukaryotic signaling pathways, which may lead to modulation of the inflammatory response, invasion of host tissue barriers, escape from immune mechanisms, or bacterial clearance. Knowledge of lipid manipulation by *P. aeruginosa* that may facilitate its persistence is essential for understanding the mechanisms underlying its pathogenicity and may provide important insights to the control of *P. aeruginosa* infections.

## Author Contributions

PC-T, AJ, LT, and AS contributed to the writing of the manuscript. All authors contributed to the article and approved the submitted version.

## Conflict of Interest

The authors declare that the research was conducted in the absence of any commercial or financial relationships that could be construed as a potential conflict of interest.

## Publisher’s Note

All claims expressed in this article are solely those of the authors and do not necessarily represent those of their affiliated organizations, or those of the publisher, the editors and the reviewers. Any product that may be evaluated in this article, or claim that may be made by its manufacturer, is not guaranteed or endorsed by the publisher.
